# Immune Evasion Mechanism and AXL

**DOI:** 10.3389/fonc.2021.756225

**Published:** 2021-10-28

**Authors:** Hye-Youn Son, Hwan-Kyu Jeong

**Affiliations:** ^1^ Department of Breast and Endocrine Surgery, Center for Medical Innovation, Seoul National University Hospital, Seoul, South Korea; ^2^ School of Biosystems and Biomedical Sciences, Korea University, Seoul, South Korea

**Keywords:** PD-1/PD-L1, Gas6/Axl signaling, immune checkpoint, immune evasion, tumor microenvironment (TME), AXL

## Abstract

Extensive interest in cancer immunotherapy is reported according to the clinical importance of CTLA-4 and (PD-1/PD-L1) [programmed death (PD) and programmed death-ligand (PD-L1)] in immune checkpoint therapies. AXL is a receptor tyrosine kinase expressed in different types of cancer and in relation to resistance against various anticancer therapeutics due to poor clinical prognosis. AXL and its ligand, i.e., growth arrest-specific 6 (GAS6) proteins, are expressed on many cancer cells, and the GAS6/AXL pathway is reported to promote cancer cell proliferation, survival, migration, invasion, angiogenesis, and immune evasion. AXL is an attractive and novel therapeutic target for impairing tumor progression from immune cell contracts in the tumor microenvironment. The GAS6/AXL pathway is also of interest immunologically because it targets fewer antitumor immune responses. In effect, several targeted therapies are selective and nonselective for AXL, which are in preclinical and clinical development in multiple cancer types. Therefore, this review focuses on the role of the GAS6/AXL signaling pathway in triggering the immunosuppressive tumor microenvironment as immune evasion. This includes regulating its composition and activating T-cell exclusion with the immune-suppressive activity of regulatory T cells, which is related to one of the hallmarks of cancer survival. Finally, this article discusses the GAS6/AXL signaling pathway in the context of several immune responses such as NK cell activation, apoptosis, and tumor-specific immunity, especially PD-1/PDL-1 signaling.

## The GAS6/AXL Signaling Pathway

Like all TYRO3, AXL, and MER (TAM) receptors, AXL is composed of two immunoglobulin-like domains, two fibronectin III (FN III) domains, a transmembrane domain, and an intracellular kinase domain (1). GAS6 is a ligand for TAM receptors, with the highest affinity for AXL (2). The gamma-carboxy glutamic (GLA) regions of GAS6 have four epithelial growth factor (EGF)-like domains and modules similar to C-terminal sex hormone-binding globulin (SHBG) that are required to activate TAM receptors. These GLA regions bind to phospholipid phosphatidylserine (3) tethered to the extracellular surface of apoptotic cells or displayed on the outer parts of photoreceptors. Phosphatidylserine stabilizes the interaction between TAM and its ligands such as GAS6 by increasing the binding affinity and slowing the rate of GAS6 dissociation from receptors (4). GAS6 activates all three receptors with different affinities (AXL > TYRO3 >>> MER) based on the ability of each ligand to activate TAM receptors (5). Upon GAS6–AXL interaction, the complex dimerizes with another GAS6–AXL complex to form a 2:2 homodimerized complex with no direct AXL/AXL or GAS6/GAS6 contacts (6). Studies have demonstrated that genetic and pharmacological inhibition of AXL affects downstream signaling pathways, which include JAK-STAT (Janus kinase/signal transducers and activators of transcription), PI3K-AKT (phosphatidylinositol 3-kinase), AKT (protein kinase B), and RAS-RAF-MEK-ERK (Rat sarcoma virus-Rapidly Accelerated Fibrosarcoma-Mitogen-activated protein kinase-Extracellular signal-regulated kinase) (7, 8).

## GAS6 and AXL Expression in the Tumor Microenvironment

The tumor microenvironment changes continuously during cancer progression by regulating oncogenic signals such as secreted factors and tumor-promoting cells to induce construction of tumor cells’ own niche (9). While AXL expression in tumors is readily recognized, it is less well known that AXL is expressed by various cells found in the tumor microenvironment, which include several immune cell types (10), fibroblasts (11), osteoclasts (12), and endothelial cells (13–15). Furthermore, the unique tumor microenvironmental conditions may modulate AXL and GAS6 expression in both tumor and immune cells to promote aggressive and pro-tumorigenic features. The tumor microenvironment can regulate AXL expression in various cells, and AXL seems to have a potential role in tumor development, progression, and metastasis.

### AXL in Host Cells

In endothelial cells, AXL expression is involved in vascularization; i.e., when it is inhibited in tumor-bearing mice, it leads to the inhibition of tumor-induced angiogenesis (16–19). The interactions between the tumor and host immune cells in the tumor microenvironment can induce the expression of AXL and GAS6 to promote a cancerous microenvironment. Tumor cells may induce the expression of AXL and GAS6 in monocytic myeloid-derived suppressor cells (M-MDSCs) and polymorphonuclear myeloid-derived suppressor cells (PMN-MDSCs) (20). Moreover, generally, AXL is expressed on bone marrow-derived cells (21–24), dendritic cells (DCs) (25, 26), macrophages (27, 28), monocytes (23), natural killer (NK) cells (29), and platelets (30).

### GAS6 in Host Cells

GAS6 is expressed by luminal progenitor and basal cells around the ductal lining of mammary tissue (31). In the bone microenvironment, GAS6 is secreted by osteoblasts, which are involved in bone formation (32, 33). It was demonstrated that osteoblast-derived GAS6 induces AXL expression in tumor cells (34), which suggests that paracrine GAS6/AXL signaling promotes survival, inhibits apoptosis, and mediates homing of tumor cells to the bone. In the tumor microenvironment, cancer-associated fibroblasts (CAFs) and CD45-expressing tumor-infiltrating leukocytes (TILs) express GAS6 (35–38), and CD45+ cells from the bone marrow or peripheral blood express significantly less GAS6 than TILs (38). Besides, macrophages and dendritic cells express high levels of GAS6 (37, 38), which can be further promoted by various cytokines (36). Especially in macrophages, *in vitro* studies suggested that tumor cells or tumor cell-conditioned media induce GAS6 expression and secretion (37, 39). Stromal cell-derived GAS6 was also shown to promote tumor cell migration, invasion, survival, and proliferation (36, 37). Potential downstream effectors of GAS6/AXL signaling through macrophage-derived GAS6 include pAKT and pSTAT3 (37).

## AXL-Mediated Tumor-Specific Immune Response

### AXL Changes Tumor Immune Microenvironment Components

During the past decades, modulating immune responses has been considered a tremendous potential therapeutics to treat cancer. Each patient’s tumor immune microenvironment (TIME) seems to be related to this treatment responsiveness. It is becoming clear that both intrinsic and extrinsic factors modulate the composition of the TIME. Specifically, several immune cells in TIME have been reported to support tumor cells’ survival through immune-suppressive functions (40, 41). Furthermore, cancer cells alter the expression of cell surface molecules to avoid detection by residential immune cells.

Several studies have revealed that GAS6/AXL signaling plays a vital role in promoting the immune-suppressive tumor microenvironment. Firstly, this signaling alters the expression level of several factors, including major histocompatibility complex I (MHC-I) and programmed death ligand-1 (PD-L1) in neoplastic cells (42). However, detailed changes are different depending on cell types and research conditions. Lung adenocarcinoma cell lines (PC9 and H1975) subjected to AXL inhibition by either bemcentinib or BGB324 significantly decreased PD-L1 (42). Pharmacologic AXL inhibition using a selective AXL inhibitor (R428 or SGI-7079) in tumor cells of C57BL/6 mice significantly increased the expression of PD-1 and MHC-I molecules (43). TAM knockout mice had increased MHC-I expression of myeloid cells. In the MCF10A cells, overexpression of TAMs did not increase PD-L1 expression, but in the PD-L1–expressing MDA-MB-231 cells, treating GAS6 liposomes increased PD-L1 expression and induced AXL phosphorylation (42). Hence, further studies are warranted to understand the detailed mechanism.

**Figure 1:**
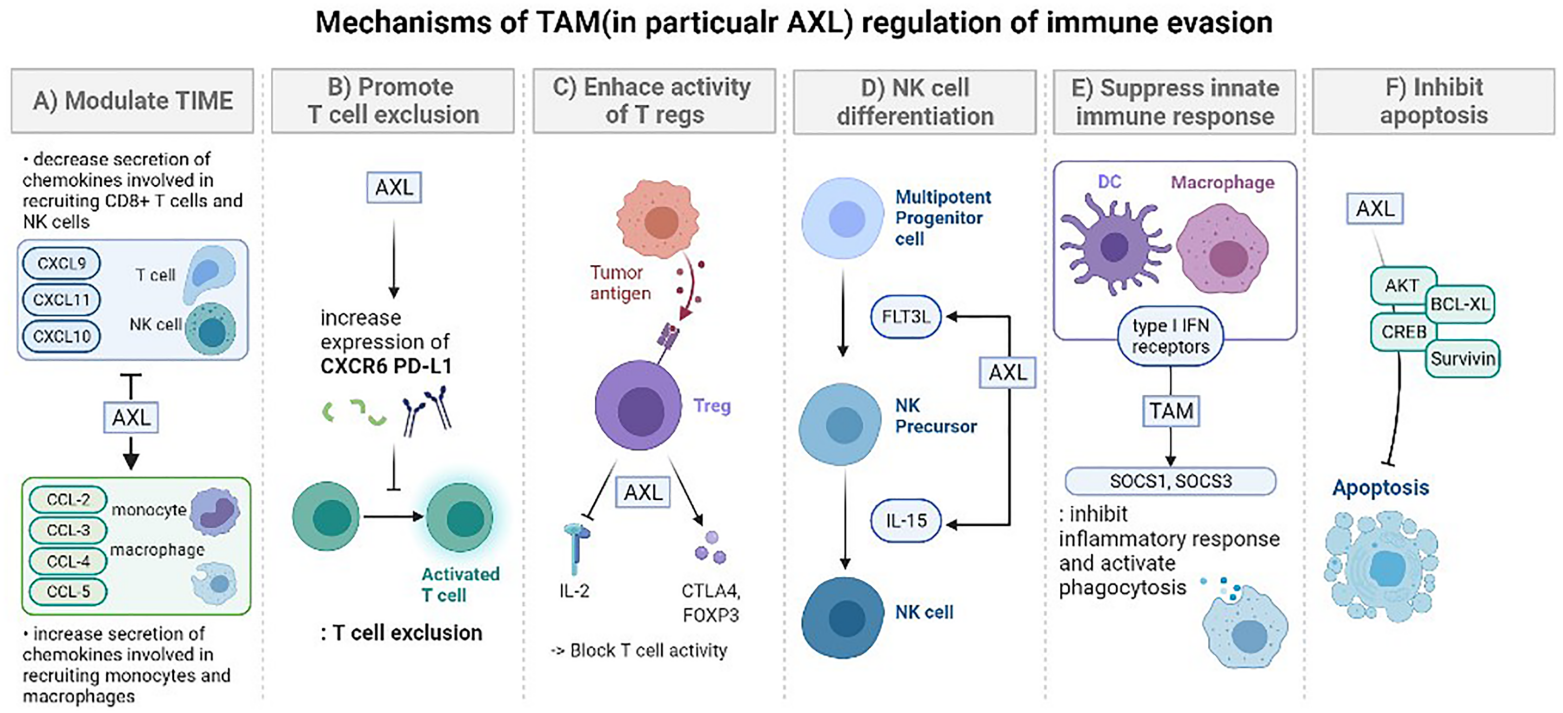
The mechanisms of TAM (in particular AXL) regulation of immune evasion. **(A)** Modulation of the tumor-immune microenvironment: promotes secretion of various immunosuppressive chemokine. After AXL inhibition, cytokines are decreased (CXCL9, CXCL10, CXCL11), increased (CCL-2, CCL-3, CCL-4, CCL5, CXCL1, CXCL2, CXCL5), or there could be no difference (CXCL12). These changes lead to regulation in the recruitment of specific immune cells (monocytes, macrophages, CD8+ T cells, NK cells). **(B)** Promoting T cell exclusion. AXL expression is significantly correlated with the expression of genes encoding CXC chemokine receptor 6 (CXCR6) and PD-L1 which prevent T cell activation. Tumors treated with the combination of pharmacological inhibition of AXL and anti-PD-1 presented an increased number of CD8 T cells. **(C)** Enhancing the immune suppressive activity of regulatory T cells (Tregs). Tumor-specific Tregs can suppress antitumor immune responses against a broad range of tumor antigens, even after being activated by just one tumor-associated antigen. GAS6 induces CD4+ CD25+ Tregs to express CTLA-4 and Foxp3 especially with AXL. These activated Tregs increase the consumption of IL-2 or suppression of IL-2 production to block the activity of T lymphocytes. **(D)** TAM signaling is involved in the overall stage of NK cell differentiation. Especially, the GAS6/AXL pathway promotes FLT3 ligand-induced human NK cell development and cooperative interaction between the GAS6/AXL pathway and IL-15 signaling promotes NK cell differentiation. In the absence of AXL, IL-15 failed to activate several downstream signaling pathways, including PI3K, AKT, and ERK1/2. **(E)** Suppress innate immune response. TAM signaling and type I IFN receptors inhibit the inflammatory response in macrophages and dendritic cells (DCs) by expressing the genes encoding the cytokine suppressors SOCS1 and SOCS3. In case of DCs, they serve as “presentation platforms” for GAS6 which triggers STAT1-dependent cascade with type I IFN. TAM also activates phagocytosis of DCs and macrophages to clear apoptotic cell corpses which could induce immune responses. **(F)** Inhibit apoptosis. The GAS6/AXL pathway is important in limiting apoptosis which involves activating survival signaling mediated by AKT, CREB, BCL-XL, and Survivin. It also suppresses phosphorylation of BAD that initiates apoptosis and activate ERK1/2 signaling.

**Figure 2:**
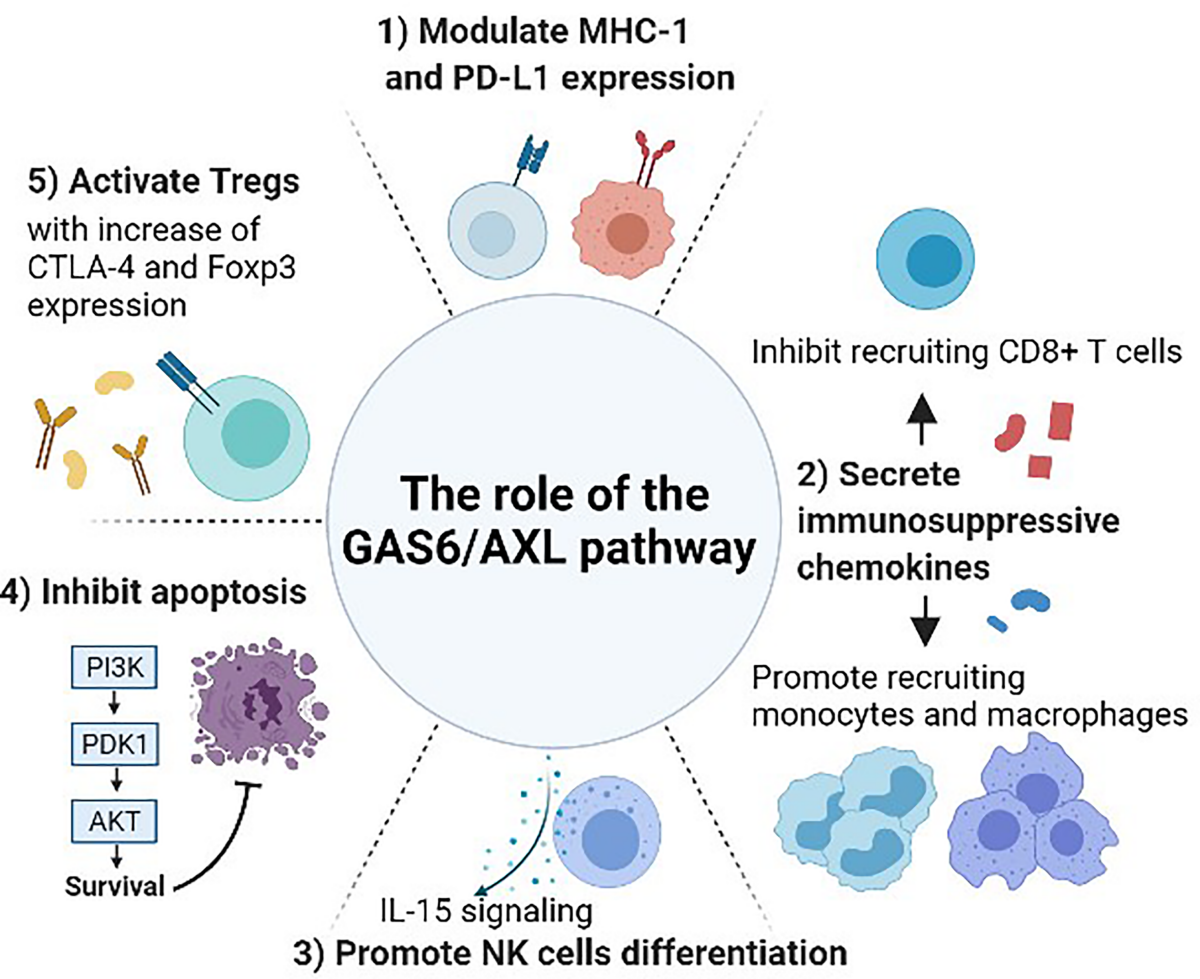
The role of the GAS6/AXL pathway between cancer and immune system. Gas6 promotes Axl expression in cancer cells, and the Gas6/Axl signaling affects tumor cell migration, invasion, survival, and proliferation. After forming the homodimerized complex, the Gas6/Axl signaling affects several downstream effectors including pAkt and pStat3 that lead to several changes in the tumor microenvironment and immune system. 1) Modulate the expression of MHC-1 and PD-L1. 2) Regulate the secretion of chemokines that are involved in recruiting several immune cells including monocytes, macrophages, and CD8+ T-cells. 3) Promote NK cell development through IL-15 signaling. As interruption of the GAS6/AXL pathway resulted in a reduction of FLT3 phosphorylation, this pathway may induce differentiation of NK cells by positively regulating FLT3 activation. 4) Inhibit apoptosis and activate phagocytosis involving several other pathways including activating the Akt and PI3K pathways. 5) Increase the immune-suppressive effect of Tregs. GAS6-induced CTLA-4 and Foxp3 expression are abrogated by blocking AXL.

Next, the GAS6/AXL signaling pathway is involved in the recruitment of both myeloid and lymphoid lineage cells, which are involved in innate and adaptive immune responses, respectively (40). It promotes the secretion of immunosuppressive cytokines, including CCL3-5, G-CSF, and TGFβ (44, 45), that are involved in the infiltration of several immune cells [macrophages and myeloid-derived suppressor cells (MDSCs)], which make it possible to escape immune surveillance (42) (**Figure 1**). Myeloid-derived suppressor cells have a certain role regarding angiogenesis, cell invasion, metastasis, and suppression of CD8+ T cells (46, 47), and the number of these cells is reduced along with AXL knockdown (48). Lymphoid lineage cells including T cells, B-cells, and NK cells are increased when pharmacologic and genetic inhibitions of AXL are treated to cancer cells (42) (**Figure 2**). However, detailed parts are still to be further demonstrated because the number of tumor-infiltrating CD8+ T-cells is increased after AXL inhibition (49), while the other research showed that AXL inhibition does not affect the number of them (21, 46, 50).

### AXL Contributes to T-Cell Exclusion

In addition to regulating TIME components, AXL also regulates T cell activity at different points. Usually, this helps control the excessive inflammatory response to protect normal cells; however, tumor cells take advantage of this protective mechanism by eliminating the T cell immune response toward cancer cells (51). In the study, not only the number of CD4+ and CD8+ T cells significantly increased, but also the gene expression associated with type 1 T-cell recruitment and functionality enhanced when AXL is inhibited in R428-treated tumor-bearing mice (43). Mainly, AXL receptor tyrosine kinase plays a particular role in T cell exclusion. AXL increases tumor cell invasion and metastasis by promoting T cell exclusion, acting as an inducer of tumor cell plasticity (52, 53). Genetic deletion of AXL resulted in up to 20-fold enhanced T-cell infiltration and sensitization of tumor cells to radiotherapy and checkpoint immunotherapy of a transgenic mouse model (50).

### Relationship Between AXL and Programmed Death 1

One of the immune checkpoints that are related to T cells is programmed death 1 (PD-1) and its ligand programmed cell death ligand 1 (PD-L1) (54). PD-L1 is expressed in several tumor cell types, and the interaction between PD-L1 and its receptor activates signaling pathways to prevent T-cell activation (55). Specifically, the expression of PD-L1 can serve as a dynamic mechanism for escaping host immune responses (56). For instance, neoplastic cells expressing PD-L1 have been reported to avoid cell death and continue to proliferate in the tumor microenvironment (42).

This PD-L1 immune checkpoint strongly interacts with AXL as AXL inhibition affects the PD-L1 pathway and activates the antitumor effect (43). When AXL is suppressed, the level of PD-L1 is decreased in lung adenocarcinoma and human triple-negative breast cancer cell lines (57, 58). However, tumor-infiltrating CD8+ T and CD4+ T cells subjected to AXL inhibition showed a noticeable induction of PD-1 on their surface. This relationship seems to be a systemic long-term memory immune response to tumor antigens (43). Furthermore, combining pharmacological inhibition of AXL with anti-PD-1 in a preclinical model of breast cancer reduces the primary tumor and metastatic burden, which is not shown when only one of them is treated. Tumors treated with the combination of these two therapeutic agents presented an increased number of CD8 T cells, with more activation of the NK cells (59). Moreover, other studies have linked AXL/PI3k signaling with increased expression of PD-L1 by tumor cells, and AXL inhibition potentiates PD-1 blockade in ID8 graft models (43, 60). This interaction is also demonstrated in lung adenocarcinoma, which showed that AXL expression significantly correlated with the expression of genes encoding PD-L1 and CXC chemokine receptor 6 (CXCR6) (57). Therefore, AXL receptor kinase is highly related to the PD-L1 immune checkpoint, contributing to immune evasion.

This relationship between AXL and PD-L1 may involve several immune cells for reactions. The analysis of TAM expression within the activated lymphoid compartment revealed that MERTK, but not AXL or TYRO3, is expressed on activated B lymphocytes and CD4+ and CD8+ T cells (61). Therefore, after apoptotic cells display PD-L1, other immune cells such as dendritic cells or macrophages sense PD-L1 involving AXL (4), and this signaling could be transferred to T-cells to take action toward immune evasion.

## Regulatory T Cells’ Immune Evasion With AXL

### Modulation of Immune Evasion With the Immune-Suppressive Activity of Regulatory T Cells

Regulatory T cells (Tregs) mediate immune evasion, which is considered a major mechanism of escaping immune surveillance (62). Especially, tumor-derived Tregs have a relatively more effective suppressive activity than naturally occurring Tregs (63, 64). These Tregs are guided to the tumor microenvironment by tumor cell-mediated chemokine production (65, 66). After that, Tregs suppress many physical and pathological immune responses, which are crucial in sustaining self-tolerance and immune homeostasis (67).

### Contribution of Treg to Immune Suppression Through Antigen

Naturally occurring Tregs are produced in the thymus and occupy 5%–10% of the total CD4 + T cells in the peripheral blood, and induced Tregs derive from naive T cells under certain conditions (68). The functional importance of Tregs in a tumor-bearing host is shown in murine models of melanoma in which depleting Tregs temporarily induce an immune response against tumors and improve tumor clearance (69).

The generation and maintenance of Tregs to regulate autoimmunity require target antigens and T-cell receptor activation (70). Natural and induced Tregs independently contribute to tumor-specific tolerance. In the case of naturally occurring Tregs, an extensive unrestricted αβ repertoire specific for a broad range of self-antigens, including tumor-associated antigens, is required which implies that the cells exercise their effect in an antigen-non-specific manner (71). One study on mice showed that induction of antigen-specific Tregs from naive cells in the tumor microenvironment does not seem to be intrinsically related to naturally occurring Tregs (72). These induced Tregs seemed to profoundly inhibit T-cell responses against tumors in an antigen-non-specific manner after being activated by a specific antigen (67). This implies that tumor-specific Tregs can suppress both medically induced and naturally occurring antitumor immune responses against a broad range of tumor antigens, even after being activated by just one tumor-associated antigen (62).

### Controlling the Effect of Tregs by GAS6, Especially With the AXL Receptor

The suppressive effect of Tregs is increased by GAS6 mainly through the AXL receptor (5). Comparing how much the GAS6-induced CTLA-4 and Foxp3 expression in CD4+ CD25+ Tregs is abrogated, blocking the AXL receptor was more effective than blocking TYRO3 and MER. In addition, GAS6 has a stronger binding affinity to AXL than TYRO3 and MER (2, 73). Therefore, GAS6 has a direct role in the functions of Tregs, which enhances the suppressive activity mostly with the AXL receptor. These interactions gain credence through IL-2, a potent T cell growth factor. When CD4+CD25-T cells pretreated with GAS6 protein are cocultured with CD4+CD25+Tregs, the proliferative activity of T cells was significantly decreased with consistent suppression of IL-2. Also, the elevated expression of CTLA-4 and Foxp3 in Tregs after Gas6 stimulation was abrogated after Axl knockdown by siRNA, and this group also showed an IL-2 level increase. These results indicate that Gas6 enhances suppression of CD4+T cells by increasing Tregs’ ability to consume IL-2 or suppress IL-2 production (74).

## The Role of TAM in NK Cell Activation Focusing on AXL and IL-15

The innate immune system is usually known to recognize pathogens directly, but it also senses and destroys cells infected with pathogens. This arm of the innate response is mainly conducted by NK cells (75). Hematopoietic stem cells (HSCs) from bone marrow differentiate into common lymphoid progenitors and develop into NK cells, followed by maturation into NK cells (76, 77). Several studies have shown that NK cells are related to tumor progression in several ways, including immune evasion. Pre-metastatic niches are promoted by suppressing NK cell functions under hypoxia (78).

### TAM Controls Natural-Killer-Cell Activation, Especially at the Differentiation Stage

TAM signaling plays a pivotal role in regulating the activity of NK cells (79). When NK cells are activated, they kill their targets by secreting the CD95 ligand and TNF-related apoptosis-inducing ligand (80–84), but NK cells from TAM-deficient mice have inferior cytotoxic activity (4). Mice lacking TAM possessed NK cells that have a defective function in both IFN-γ production and cytotoxicity. This activity impairment is proportional to how effectively TAM genes are inactivated as all three TAM receptors are expressed by immature NK cells in the bone marrow (79, 85). The number of NK cells generated from human CD34+ HPCs reduced after blocking GAS6 binding to AXL by AXL-Fc or warfarin (86).

After proving that TAM regulates NK cell activity, the specific stage that TAM is mainly involved in is considered. When immature cells are grown with NIH3T3 fibroblasts expressing GAS6, stromal cells can restore their ability to drive NK-cell maturation *in vitro*. Furthermore, the mice showed normal perforin and granzyme B levels even when NK cells lack all three TAM receptors (4). However, these cells do not secrete IFNγ, which is produced predominantly by NK cells and exhibit a 10-fold lower killing ability against target cells than wild-type NK cells after stimulation. This means that these cells do not fully demonstrate the expression of activation and inhibition receptors expressed by cytotoxic NK cells (4). Thus, TAM signaling is involved in the terminal stage of NK cell differentiation.

### Importance of Interleukin-15 and AXL in NK Cell Activation

Interleukin-15 (IL-15) is another critical factor for NK cell development, which contributes to the differentiation, survival, and function of NK cells (87). When the mice are deficient in Interleukin-2 (IL-2) and IL-15 receptor, which is required only for the actions of IL-2 and IL-15 rather than other growth factors, NK cells are deficient. However, a normal level of NK cells is observed in mice deficient in IL-2 or IL-2Rα, the private receptor used by IL-2, suggesting that IL-15 is an essential factor in the differentiation of NK cells from uncommitted progenitors (88). Furthermore, the upregulation of NK cell activity was markedly reduced by the addition of monoclonal antibodies to IL-15, but not by antibodies to other cytokines such as IFN-α, IFN-γ, TNF-α, TGF-β, and IL-2 (89). This evidence supports the suggestion that IL-15 is essential in the activation of NK cells.

Several studies have shown that the IL-15 and GAS6/AXL pathways partially overlap intracellular signaling molecules (90). In the absence of AXL, IL-15 failed to activate several downstream signaling pathways, including PI3K, AKT, and ERK1/2 (91).

### Enhancing the Role of AXL and IL-15 Through FMS-Like Tyrosine Kinase 3

The link between IL-15 and AXL could be extended to FMS-like tyrosine kinase 3 (FLT3), one of the receptor tyrosine kinases (RTK). Mice with genetic disruption at the FLT3 locus presented a reduction of the numbers of B-lymphoid progenitors, dendritic cells, and NK cells (92). The FLT3 ligand can enhance NK cell differentiation in the presence of IL-15 (93). To activate the FLT3 and its ligand (FL) pathway, the phosphorylation of FLT3 is essential, which follows the binding of its ligand FL (94, 95). Interruption of the GAS6/AXL pathway resulted in a marked reduction of FLT3 phosphorylation even in the presence of FL. This implies that the GAS6/AXL pathway promotes FL-induced human NK cell development by positively regulating FLT3 activation (96) (**Figure 1**). As FLT3 can induce differentiation of NK cells with IL-15 and the interaction between AXL and FLT3 is demonstrated, AXL and IL-15 could be highly related to each other in various immune cell activation steps.

## Regulation of Innate Immune Cells by AXL

In addition to its role in NK cell activation, TAM has another role in regulating the innate immune response. TAM signaling is generally activated by Toll-like receptor (TLR) and type I interferon signaling, which is part of the innate inflammatory response in dendritic cells (DCs) and macrophages (5). The AXL receptor was found to be upregulated when DCs are cultured with type I IFNs (10, 26, 97). Additionally, the co-expression of TAM and type I IFN receptors in macrophages and dendritic cells (DCs) inhibits the inflammatory response of the innate immune system through induction of the genes encoding the cytokine suppressors SOCS1 and SOCS3. More specifically, the binding of the apoptotic cells to DCs is immunosuppressive and serves as “presentation platforms” for GAS6. This triggers a STAT1-dependent cascade with type I IFN, and this signaling also induces SOCS1 and SOCS3 expressions, which inhibit downstream signaling pathways of TLRs and cytokine receptors (5). Thus, the innate immune mechanism is proceeded and dependent on TAM receptors.

## Regulation of Immune Evasion by AXL Through Apoptosis and Phagocytosis

Several pieces of research have shown the relationship between AXL and apoptosis. Apoptosis produces materials that can induce an immune response, which should be prevented for tumor immune evasion.

### Inhibition of Apoptosis by GAS6 With AXL Kinase

Apoptosis of vascular smooth muscle cells (VSMCs) has been identified in the physiological remodeling of the vasculature. Cell death with cell proliferation, migration, and matrix turnover may induce changes in vascular architecture during development and diseases such as atherosclerosis (98). This apoptosis of VSMCs is coupled with GAS6 binding to the AXL receptor, and GAS6 inhibits apoptosis in cultured VSMCs through AXL phosphorylation (99). GAS6 and AXL are increased after vascular injury, and these molecules play an essential role in neointima formation by suppressing apoptosis (100). Besides, it is speculated that the GAS6/AXL pathway is vital to limiting VSMC apoptosis by activating AKT and PI3K along with several other pathways, including phosphorylation of BAD (BCL2-associated agonist of cell death) and activation of ERK1/2 (101–103).

Non-small cell lung cancer (NSCLC), a prevalent and devastating disease, shows overexpression and activation of MER and AXL. MER or AXL knockdown also improved *in vitro* NSCLC sensitivity to chemotherapeutic agents by promoting apoptosis. Also, AXL inhibition induces apoptosis by abating survival signaling mediated by AKT, CREB, BCL-XL, and survivin (104).

### Removal of Apoptosis Remains by Activating Phagocytosis

Furthermore, some cancers develop specific mechanisms to clear apoptotic cells to regulate immune responses. Defects in the clearance of apoptotic cells can induce an immune response. Macrophages and DCs must remove many apoptotic cells corpses, but this form of homeostatic phagocytosis is impaired in TAM-deficient mice (24, 105, 106). This immunosuppressive effect of DCs is exerted through TAM signaling, and this immune response may be reinforced, especially in cancer cells as they overexpress AXL. Hence, TAM contributes to immune evasion by activating phagocytosis to remove these remains.

However, there is a possibility that TAM signaling induced by apoptotic cells is autocrine which means macrophages and DCs themselves produce GAS6, not from the apoptotic cells. In this case, phosphatidylserine is the primary stimulant that stabilized the interaction between TAM and its ligands (4). Thus, further research is needed to understand the exact mechanism.

## Clinical Trial Suppressing AXL

Targeting AXL for cancer treatment is under the spotlight, and several clinical studies involving the use of anti-AXL have been conducted. The first clinical trial treating an anti-AXL-specific small molecule inhibitor called BGB324 was performed in 2013. BGB324 blocks auto-phosphorylation of AXL on the COOH-terminal multiple docking sites Tyr821 with the subsequent activation of AKT and SFK phosphorylation (107). After entering phase I clinical trials in 2013, it is currently under phase II study to assess the safety of BGB324 when given in up to 77 patients advanced adenocarcinoma of the lung previously treated with pembrolizumab (108). Since then, several newly synthesized inhibitors specific for AXL receptors are being tested at a clinical stage.

Many small-molecule inhibitors in the clinical stage do not solely target AXL. Some inhibitors work as AXL pathway modulators that target factors such as MET, TYRO3, and FLT3 along with AXL and are used with several immune checkpoints inhibitors (ICIs), including nivolumab, pembrolizumab, durvalumab, and avelumab (109).

Small molecules are being developed mainly, and other therapeutic agents such as a monoclonal antibody or nucleotide aptamer are also in preclinical progress. The YW327.6S2 phage-derived monoclonal antibody binds to human AXL with high affinity, which blocks the GAS6 binding to the receptor and downregulates receptor expression (110). Aptamers are short structured single-stranded RNAs or DNAs that bind to a specific target molecule. They are promising alternatives with great potentials because of their low cost, lower toxicity, and higher affinity (111, 112). A selective RNA-based aptamer, GL21.T, binds the extracellular domain of AXL at high affinity and inhibits its catalytic activity. This includes ERK and AKT phosphorylation and inhibited *in vivo* lung tumor formation in mouse xenografts (111) (**Table 1**).

**Table 1:** AXL inhibitor under development.

Compound	Target	Phase of development	Sponsor
**< Small-molecule inhibitors > (** 113 **)**			
**ASP2215**(Gilteritinib)	AXL, FLT3, ALK	Phase 3 (acute myeloid leukemia)	Astellas Pharma.
**BGB324**(R428)	AXL (selective)	Phase 2 (triple-negative breast cancer, lung cancer metastatic)	Rigel PharmaceuticalsBerGenBIO
**BMS-777607** (ASLAN002)	AXL, RON, MET, TYRO3, MER, FLT3	Phase 2 (advanced solid tumors)	Aslan Pharma. and Inventive Health Clinical
**DP3975**	AXL	Preclinical	Deciphera Biotech
**GSK1363089**/XL880 (Foretinib)	AXL, MET, VEGFR2, RON	Phase 2 (breast cancer/carcinoma, renal cell etc.)	GlaxoSmithKline
**LDC1267**	MET, AXL, TYRO3	Preclinical	Lead Discovery Centre
**LY2801653**(Merestinib)	MET, MSTIR, DDRI, TIEI, MER, TYRO3, AXL	Phase 2 (carcinoma, non-small-cell lung/active, not recruiting)	Eli Lilly and Co.
**MGCD 265**	MET, AXL, VEGFR2	Phase 2 (non-small cell lung cancer)Phase 2 (carcinoma, non-small-cell lung/active, not recruiting)	Mirati Inc.
**MGCD516**(Sitravatinib)	MET, AXL, RET, TRK, DDR2	Phase 3 (non-small cell lung cancer, advanced or metastatic solid malignancies)	Mirati Inc.
**MP-470**(Amuvatinib)	KIT, PDGFR1, FLT3, RET, AXL	Phase 1 (solid tumors)Phase 2 (small cell lung carcinoma)	Astex Pharma.
**NPS-1034**	AXL, DDR1, FLT3, KIT, MEK, MET, ROS1, TIE1	Preclinical	NeoPharma
**PF-02341066**(Crizotinib)	ALK, MET, RON, AXL	Approved for non-small-cell lung cancerPhase 2 (solid tumors)	Pfizer, NCI, EORTC etc.
**SGI-7079**	MET, MER, YES, RET, FLT3, AXL	Preclinical	Astex Pharma
**SKI-606**(Bosutinibm)	BCR-ABL, ABL, SRC, YES, MEK, AXL, BMX	Phase 3 (chronic myeloid leukemia/recruiting)Phase 2 (breast neoplasms)	Pfizer
**SU11248**(Sunitinib)	KIT, FLT3, PDGFR, VEGFR2, AXL	Approved for renal cell carcinoma, imatinib-resistant gastrointestinal stromal tumor, and metastatic pancreatic neuroendocrine tumorsPhase 3 (different solid tumors)	Pfizer/Further clinical sponsors include NCI, Baylor Breast Care Center, M.D. Anderson Cancer Center, etc.
**TP-0903**	JAK2, ALK, ABL, AXL, MER	Phase 2 (acute myeloid leukemia)	Huntsman Cancer Institute/Tolero Pharmaceuticals
**UNC2025**	MER, FLT3, AXL, TYRO3	Preclinical	University of North Carolina
**XL184**(Cabozantinib)	VEGFR2, MET, MEK, KIT, RET, AXL	Approved (medullary thyroid cancer)Phase 2, 3 for different solid tumors	Exelixis
**< Receptor monoclonal antibody >**			
**YW327.6S2**	AXL	Preclinical (non-small cell lung cancer)	(111)
**D9**	AXL	Preclinical (pancreatic cancer)	(114)
**E8**	AXL	Preclinical (pancreatic cancer)	(114)
**< Nucleotide aptamer >**			
**GL21.T**(RNA apatamer)	AXL	Preclinical (non-small cell lung cancer)	(111)
**DNA AXL-Apatamar**	AXL	Preclinical (ovarian cancer)	(17)

## Author Contributions

H-YS contributed to the conception and design of the study. H-YS and H-KJ wrote sections of the manuscript. All authors contributed to the manuscript revision and read and approved the submitted version.

## Funding

This work was supported by the grant no. 04-2021-0570 from SNUH Research Fund.

## Conflict of Interest

The authors declare that the research was conducted in the absence of any commercial or financial relationships that could be construed as a potential conflict of interest.

## Publisher’s Note

All claims expressed in this article are solely those of the authors and do not necessarily represent those of their affiliated organizations, or those of the publisher, the editors and the reviewers. Any product that may be evaluated in this article, or claim that may be made by its manufacturer, is not guaranteed or endorsed by the publisher.

## References

[1] O’Bryan JP Frye RA Cogswell PC Neubauer A Kitch B Prokop C. Axl, a Transforming Gene Isolated From Primary Human Myeloid Leukemia Cells, Encodesa Novel Receptor Tyrosine Kinase. Mol Cell Biol (1991) 11:5016–31. doi: 10.1128/mcb.11.10.5016-5031.1991 PMC361494 1656220

[2] Nagata K Ohashi K Nakano T Arita H Zong C Hanafusa H. Identification of the Product of Growth Arrest-Specific Gene 6 as a Common Ligand for Axl, Sky, and Mer Receptor Tyrosine Kinases. J Biol Chem (1996) 271:30022–7. doi: 10.1074/jbc.271.47.30022 8939948

[3] Huang M Rigby AC Morelli X Grant MA Huang G Furie B. Structural Basis of Membrane Binding by Gla Domains of Vitamin K–dependent Proteins. Nat Struct Mol Biol (2003) 10:751–6. doi: 10.1038/nsb971 12923575

[4] Lemke G Rothlin CV. Immunobiology of the TAM Receptors. Nat Rev Immunol (2008) 8.5:327–36. doi: 10.1038/nri2303 PMC2856445 18421305

[5] Rothlin CV Greg L. TAM Receptor Signaling and Autoimmune Disease. Curr Opin Immunol (2010) 22(6):740–6. doi: 10.1016/j.coi.2010.10.001 PMC2997887 21030229

[6] Sasaki T Knyazev PG Clout NJ Cheburkin Y Gohring W Ullrich A. Structural Basis for Gas6-Axl Signalling. EMBO J (2006) 25:80–7. doi: 10.1038/sj.emboj.7600912 PMC1356355 16362042

[7] Gay CM Balaji K Byers LA. Giving AXL the Axe: Targeting AXL in Human Malignancy. Nat Publ Group (2017) 116:415–23. doi: 10.1038/bjc.2016.428 PMC5318970 28072762

[8] Cruz VH Arner EN Du W Bremauntz AE Brekken RA. Axl-Mediated Activation of TBK1 Drives Epithelial Plasticity in Pancreatic Cancer. JCI Insight (2019) 4(9):e126117. doi: 10.1172/jci.insight.126117 PMC6538328 30938713

[9] Quail DF Joyce JA. Microenvironmental Regulation of Tumor Progression and Metastasis. Nat Med (2013) 19:1423–37. doi: 10.1038/nm.3394 PMC3954707 24202395

[10] Rothlin CV Ghosh S Zuniga EI Oldstone MB Lemke G. TAM Receptors are Pleiotropic Inhibitors of the Innate Immune Response. Cell (2007) 131:1124–36. doi: 10.1016/j.cell.2007.10.034 18083102

[11] Espindola MS Habiel DM Narayanan R Jones I Coelho AL Murray LA. Targeting of TAM Receptors Ameliorates Fibrotic Mechanisms in Idiopathic Pulmonary Fibrosis. Am J Respir Crit Care Med (2018) 197:1443–56. doi: 10.1164/rccm.201707-1519OC PMC6005556 29634284

[12] Nakamura YS Hakeda Y Takakura N Kameda T Hamaguchi I Miyamoto T. Tyro 3 Receptor Tyrosine Kinase and its Ligand, Gas6, Stimulate the Function of Osteoclasts. Stem Cells (1998) 16:229–38. doi: 10.1002/stem.160229 9617898

[13] Gallicchio M Mitola S Valdembri D Fantozzi R Varnum B Avanzi GC. Inhibition of Vascular Endothelial Growth Factor Receptor 2-Mediated Endothelial Cell Activation by Axl Tyrosine Kinase Receptor. Blood (2005) 105:1970–6. doi: 10.1182/blood-2004-04-1469 15507525

[14] Fedeli C Torriani G Galan-Navarro C Moraz ML Moreno H Gerold G. Axl Can Serve as Entry Factor for Lassa Virus Depending on the Functional Glycosylation of Dystroglycan. J Virol (2018) 92(5):e01613–17. doi: 10.1128/JVI.01613-17 PMC5809728 29237830

[15] Holland SJ Powell MJ Franci C Chan EW Friera AM Atchison RE. Multiple Roles for the Receptor Tyrosine Kinase Axl in Tumor Formation. Cancer Res (2005) 65:9294–303. doi: 10.1158/0008-5472.CAN-05-0993 16230391

[16] Lei X Chen M Nie Q Hu J Zhuo Z Yiu A. *In Vitro* and *In Vivo* Antiangiogenic Activity of Desacetylvinblastine Monohydrazide Through Inhibition of VEGFR2 and Axl Pathways. Am J Cancer Res (2016) 6(4):843–58. PMC4859888 27186435

[17] Kanlikilicer P Ozpolat B Aslan B Bayraktar R Gurbuz N Rodriguez-Aguayo C. Therapeutic Targeting of AXL Receptor Tyrosine Kinase Inhibits Tumor Growth and Intraperitoneal Metastasis in Ovarian Cancer Models. Mol Nucleic Acids (2017) 9:251–62. doi: 10.1016/j.omtn.2017.06.023 PMC5675720 29246304

[18] Xiao Y Zhao H Tian L Nolley R Diep AN Ernst A. S100A10 Is a Critical Mediator of GAS6/AXL-Induced Angiogenesis in Renal Cell Carcinoma. Cancer Res (2019) 79:5758–68. doi: 10.1158/0008-5472.CAN-19-1366 PMC7015045 31585940

[19] Tanaka M Siemann DW. Axl Signaling is an Important Mediator of Tumor Angiogenesis. Oncotarget (2019) 10:2887–98. doi: 10.18632/oncotarget.26882 PMC6499597 31080559

[20] Holtzhausen A Harris W Ubil E Hunter DM Zhao J Zhang Y. TAM Family Receptor Kinase Inhibition Reverses MDSC-Mediated Suppression and Augments Anti-PD-1 Therapy in Melanoma. Cancer Immunol Res (2019) 7:1672–86. doi: 10.1158/2326-6066.CIR-19-0008 PMC6943983 31451482

[21] Huey MG Minson KA Earp HS DeRyckere D Graham DK. Targeting the TAM Receptors in Leukemia. Cancers (Basel) (2016) 8:101. doi: 10.3390/cancers8110101 PMC5126761 27834816

[22] Kasikara C Davra V Calianese D Geng K Spires TE Quigley M. Pan-TAM Tyrosine Kinase Inhibitor BMS-777607 Enhances Anti-PD-1 mAb Efficacy in a Murine Model of Triple-Negative Breast Cancer. Cancer Res (2019) 79:2669–83. doi: 10.1158/0008-5472 30877108

[23] Neubauer A Fiebeler A Graham DK O’Bryan JP Schmidt CA Barckow P. Expression of Axl, a Transforming Receptor Tyrosine Kinase, in Normal and Malignant Hematopoiesis. Blood (1994) 84:1931–41. doi: 10.1182/blood.V84.6.1931.1931 7521695

[24] Satomura K Derubeis AR Fedarko NS Ibaraki-O’Connor K Kuznetsov SA Rowe DW. Receptor Tyrosine Kinase Expression in Human Bone Marrow Stromal Cells. J Cell Physiol (1998) 177:426–38. doi: 10.1002/(SICI)1097-4652(199812)177:3<426::AID-JCP6>3.0.CO;2-F 9808151

[25] Seitz HM Camenisch TD Lemke G Earp HS Matsushima GK. Macrophages and Dendritic Cells Use Different Axl/Mertk/Tyro3 Receptors in Clearance of Apoptotic Cells. J Immunol (2007) 178:5635–42. doi: 10.4049/jimmunol.178.9.5635 17442946

[26] Subramanian M Hayes CD Thome JJ Thorp E Matsushima GK Herz J. An AXL/LRP-1/RANBP9 Complex Mediates DC Efferocytosis and Antigen Cross-Presentation *In Vivo* . J Clin Investig (2014) 124:1296–308. doi: 10.1172/JCI72051 PMC3934164 24509082

[27] Sharif MN Sosic D Rothlin CV Kelly E Lemke G Olson EN. Twist Mediates Suppression of Inflammation by Type I IFNs and Axl. J Exp Med (2006) 203:1891–901. doi: 10.1084/jem.20051725 PMC2118370 16831897

[28] Deng T Zhang Y Chen Q Yan K Han D. Toll-Like Receptor-Mediated Inhibition of Gas6 and ProS Expression Facilitates Inflammatory Cytokine Production in Mouse Macrophages. Immunology (2012) 135:40–50. doi: 10.1111/j.1365-2567.2011.03511.x 22043818 PMC3246651

[29] Paolino M Choidas A Wallner S Pranjic B Uribesalgo I Loeser S. The E3 Ligase Cbl-B and TAM Receptors Regulate Cancer Metastasis *via* Natural Killer Cells. Nature (2014) 507:508–12. doi: 10.1038/nature12998 PMC6258903 24553136

[30] Gould WR Baxi SM Schroeder R Peng YW Leadley RJ Peterson JT. Gas6 Receptors Axl, Sky and Mer Enhance Platelet Activation and Regulate Thrombotic Responses. J Thromb Haemost (2005) 3:733–41. doi: 10.1111/j.1538-7836.2005.01186.x 15733062

[31] Mills KL Gomes AM Standlee CR Rojo MD Carmeliet P Lin Z. Gas6 is Dispensable for Pubertal Mammary Gland Development. PloS One (2018) 13(12):e0208550. doi: 10.1371/journal.pone.0208550 30533018 PMC6289431

[32] Shiozawa Y Pedersen EA Patel LR Ziegler AM Havens AM Jung Y. GAS6/AXL Axis Regulates Prostate Cancer Invasion, Proliferation, and Survival in the Bone Marrow Niche. Neoplasia (2010) 12:116–27. doi: 10.1593/neo.91384 PMC2814350 20126470

[33] Shiozawa Y Pedersen EA Taichman RS. GAS6/Mer Axis Regulates the Homing and Survival of the E2A/PBX1-Positive B-Cell Precursor Acute Lymphoblastic Leukemia in the Bone Marrow Niche. Exp Hematol (2010) 38:132–40. doi: 10.1016/j.exphem.2009.11.002 PMC2815170 19922767

[34] Khoo WH Ledergor G Weiner A Roden DL Terry RL McDonald MM. A Niche-Dependent Myeloid Transcriptome Signature Defines Dormant Myeloma Cells. Blood (2019) 134:30–43. doi: 10.1182/blood.2018880930 31023703

[35] Kanzaki R Naito H Kise K Takara K Eino D Minami M. Gas6 Derived From Cancer-Associated Fibroblasts Promotes Migration of Axl-Expressing Lung Cancer Cells During Chemotherapy. Sci Rep (2017) 7:10613. doi: 10.1038/s41598-017-10873-2 28878389 PMC5587707

[36] Bae CA Ham IH Oh HJ Lee D Woo J Son SY. Inhibiting the GAS6/AXL Axis Suppresses Tumor Progression by Blocking the Interaction Between Cancer-Associated Fibroblasts and Cancer Cells in Gastric Carcinoma. Gastric Cancer (2020) 23:824–36. doi: 10.1007/s10120-020-01066-4 32239298

[37] Gomes AM Carron EC Mills KL Dow AM Gray Z Fecca CR. Stromal Gas6 Promotes the Progression of Premalignant Mammary Cells. Oncogene (2019) 38:2437–50. doi: 10.1038/s41388-018-0593-5 PMC6450766 30531835

[38] Loges S Schmidt T Tjwa M van Geyte K Lievens D Lutgens E. Malignant Cells Fuel Tumor Growth by Educating Infiltrating Leukocytes to Produce the Mitogen Gas6. Blood (2010) 115:2264–73. doi: 10.1182/blood-2009-06-228684 19965679

[39] Carron EC Homra S Rosenberg J Coffelt SB Kittrell F Zhang Y. Macrophages Promote the Progression of Premalignant Mammary Lesions to Invasive Cancer. Oncotarget (2017) 8:50731–46. doi: 10.18632/oncotarget.14913 PMC5584199 28881599

[40] Hanahan D Weinberg RA. Hallmarks of Cancer: The Next Generation. Cell (2011) 144:646–74. doi: 10.1016/j.cell.2011.02.013 21376230

[41] Binnewies M Roberts EW Kersten K Chan V Fearon DF Merad M. Understanding the Tumor Immune Microenvironment (TIME) for Effective Therapy. Nat Med (2018) 24:541–50. doi: 10.1038/s41591-018-0014-x PMC5998822 29686425

[42] Tanaka M Siemann DW. Gas6/Axl Signaling Pathway in the Tumor Immune Microenvironment. Cancers (2020) 12(7):1850. doi: 10.3390/cancers12071850 PMC7408754 32660000

[43] Guo Z Li Y Zhang D Ma J. Axl Inhibition Induces the Antitumor Immune Response Which can be Further Potentiated by PD-1 Blockade in the Mouse Cancer Models. Oncotarget (2017) 8(52):89761–74. doi: 10.18632/oncotarget.21125 PMC5685707 29163786

[44] Dranoff G. Cytokines in Cancer Pathogenesis and Cancer Therapy. Nat Rev Cancer (2004) 4:11–22. doi: 10.1038/nrc1252 14708024

[45] Chow MT Luster AD. Chemokines in Cancer. Cancer Immunol Res (2014) 2:1125–31. doi: 10.1158/2326-6066.CIR-14-0160 PMC4258879 25480554

[46] Bronte V Wang M Overwijk WW Surman DR Pericle F Rosenberg SA. Apoptotic Death of CD8+ T Lymphocytes After Immunization: Induction of a Suppressive Population of Mac-1+/Gr-1+ Cells. J Immunol (1998) 161(10):5313–20. PMC2239007 9820504

[47] Yang L DeBusk LM Fukuda K Fingleton B Green-Jarvis B Shyr Y. Expansion of Myeloid Immune Suppressor Gr+CD11b+ Cells in Tumor-Bearing Host Directly Promotes Tumor Angiogenesis. Cancer Cell (2004) 6:409–21. doi: 10.1016/j.ccr.2004.08.031 15488763

[48] Ludwig KF Du W Sorrelle NB Wnuk-Lipinska K Topalovski M Toombs JE. Small-Molecule Inhibition of Axl Targets Tumor Immune Suppression and Enhances Chemotherapy in Pancreatic Cancer. Cancer Res (2018) 78(1):246–55. doi: 10.1158/0008-5472.CAN-17-1973 PMC5754222 29180468

[49] Hua K-T Liu Y-F Hsu C-L Cheng T-Y Yang C-Y Chang J-S. 30UTR Polymorphisms of Carbonic Anhydrase IX Determine the miR-34a Targeting Efficiency and Prognosis of Hepatocellular Carcinoma. Sci Rep (2017) 7:4466. doi: 10.1038/s41598-017-04732-3 28667334 PMC5493636

[50] Aguilera TA Rafat M Castellini L Shehade H Kariolis MS Hui AB. Reprogramming the Immunological Microenvironment Through Radiation and Targeting Axl. Nat Commun (2016) 7:13898. doi: 10.1038/ncomms13898 28008921 PMC5196438

[51] De Sousa Linhares A Leitner J Grabmeier-Pfistershammer K Steinberger P. Not All Immune Checkpoints Are Created Equal. Front Immunol (2018) 9:1909. doi: 10.3389/fimmu.2018.01909 30233564 PMC6127213

[52] Paolino M Penninger JM. The Role of TAM Family Receptors in Immune Cell Function: Implications for Cancer Therapy. Cancers (Basel) (2016) 8(10):97. doi: 10.3390/cancers8100097 PMC5082387 27775650

[53] Aguilera TA Giaccia AJ. Molecular Pathways: Oncologic Pathways and Their Role in T-Cell Exclusion and Immune Evasion-a New Role for the AXL Receptor Tyrosine Kinase. Clin Cancer Res (2017) 23:2928–33. doi: 10.1158/1078-0432.CCR-17-0189 PMC5474155 28289089

[54] Ishida Y Agata Y Shibahara K Honjo T. Induced Expression of PD-1, a Novel Member of the Immunoglobulin Gene Superfamily, Upon Programmed Cell Death. EMBO J (1992) 11:3887–95. doi: 10.1002/j.1460-2075.1992.tb05481.x PMC556898 1396582

[55] Keir ME Liang SC Guleria I Latchman YE Qipo A Albacker LA. Tissue Expression of PD-L1 Mediates Peripheral T Cell Tolerance. J Exp Med (2006) 203:883–95. doi: 10.1084/jem.20051776 PMC2118286 16606670

[56] Iwai Y Ishida M Tanaka Y Okazaki T Honjo T Minato N. Involvement of PD-L1 on Tumor Cells in the Escape From Host Immune System and Tumor Immunotherapy by PD-L1 Blockade. Proc Natl Acad Sci U S A (2002) 99:12293–7. doi: 10.1073/pnas.192461099 PMC129438 12218188

[57] Tsukita Y Fujino N Miyauchi E Saito R Fujishima F Itakura K. Axl Kinase Drives Immune Checkpoint and Chemokine Signalling Pathways in Lung Adenocarcinomas. Mol Cancer (2019) 18:24. doi: 10.1186/s12943-019-0953-y 30744655 PMC6369543

[58] Kasikara C Kumar S Kimani S Tsou WI Geng K Davra V. Phosphatidylserine Recognition by Phagocytes Sensing by TAM Receptors Regulates AKT-Dependent Chemoresistance and PD-L1 Expression. Mol Cancer Res (2017) 15:753–64. doi: 10.1158/1541-7786.MCR-16-0350 PMC8363069 28184013

[59] Goyette M-A Elkholi IE Apcher C Kuasne H Rothlin CV Muller WJ. Targeting Axl Favors an Antitumorigenic Microenvironment That Enhances Immunotherapy Responses by Decreasing Hif-1α Levels. Proc Natl Acad Sci (2021) 118(29):e2023868118. doi: 10.1073/pnas.2023868118 34266948 PMC8307381

[60] Skinner HD Giri U Yang LP Kumar M Liu Y Story MD. Integrative Analysis Identifies a Novel AXL-PI3 Kinase-PD-L1 Signaling Axis Associated With Radiation Resistance in Head and Neck Cancer. Clin Cancer Res (2017) 23:2713–22. doi: 10.1158/1078-0432.CCR-16-2586 PMC5457365 28476872

[61] Giroud P Renaudineau S Gudefin L Calcei A Menguy T Rozan C. Expression of TAM-R in Human Immune Cells and Unique Regulatory Function of MerTK in IL-10 Production by Tolerogenic DC. Front Immunol (2020) 11:564133. doi: 10.3389/fimmu.2020.564133 33101282 PMC7546251

[62] Jacobs JF Nierkens S Figdor CG de Vries IJ Adema GJ. Regulatory T Cells in Melanoma: The Final Hurdle Towards Effective Immunotherapy? Lancet Oncol (2012) 13:e32–42. doi: 10.1016/S1470-2045(11)70155-3 22225723

[63] Yokokawa J Cereda V Remondo C Gulley JL Arlen PM Schlom J. Enhanced Functionality of CD4+CD25(high)FoxP3+ Regulatory T Cells in the Peripheral Blood of Patients With Prostate Cancer. Clin Cancer Res (2008) 14:1032–40. doi: 10.1158/1078-0432.CCR-07-2056 18281535

[64] Gasparoto TH de Souza Malaspina TS Benevides L de Melo EJ Jr Costa MR Damante JH. Patients With Oral Squamous Cell Carcinoma are Characterized by Increased Frequency of Suppressive Regulatory T Cells in the Blood and Tumor Microenvironment. Cancer Immunol Immunother (2010) 59:819–28. doi: 10.1007/s00262-009-0803-7 PMC11030726 20012605

[65] Curiel TJ Coukos G Zou L Alvarez X Cheng P Mottram P. Specific Recruitment of Regulatory T Cells in Ovarian Carcinoma Fosters Immune Privilege and Predicts Reduced Survival. Nat Med (2004) 10:942–9. doi: 10.1038/nm1093 15322536

[66] Lee I Wang L Wells AD Dorf ME Ozkaynak E Hancock WW. Recruitment of Foxp3+ T Regulatory Cells Mediating Allograft Tolerance Depends on the CCR4 Chemokine Receptor. J Exp Med (2005) 201:1037–44. doi: 10.1084/jem.20041709 PMC2213137 15809349

[67] Sakaguchi S Yamaguchi T Nomura T Ono M. Regulatory T Cells and Immune Tolerance. Cell (2008) 133:775–87. doi: 10.1016/j.cell.2008.05.009 18510923

[68] Fontenot JD Gavin MA Rudensky AY. Foxp3 Programs the Development and Function of CD4+CD25+ Regulatory T Cells. Nat Immunol (2003) 4:330–36. doi: 10.1038/ni904 12612578

[69] Nizar S Meyer B Galustian C Kumar D Dalgleish A. T Regulatory Cells, the Evolution of Targeted Immunotherapy. Biochim Biophys Acta (2010) 1806:7–17. doi: 10.1016/j.bbcan.2010.02.001 20188145

[70] Samy ET Setiady YY Ohno K Pramoonjago P Sharp C Tung KS. The Role of Physiological Self-Antigen in the Acquisition and Maintenance of Regulatory T-Cell Function. Immunol Rev (2006) 212:170–84. doi: 10.1111/j.0105-2896.2006.00404.x 16903914

[71] Fourcade J Sun Z Kudela P Janjic B Kirkwood JM El-Hafnawy T. Human Tumor Antigen-Specific Helper and Regulatory T Cells Share Common Epitope Specificity But Exhibit Distinct T Cell Repertoire. J Immunol (2010) 184:6709–18. doi: 10.4049/jimmunol.0903612 PMC7382897 20483736

[72] Zhou G Levitsky HI. Natural Regulatory T Cells and *De Novo*-Induced Regulatory T Cells Contribute Independently to Tumor-Specific Tolerance. J Immunol (2007) 178:2155–62. doi: 10.4049/jimmunol.178.4.2155 17277120

[73] Sinha S Boysen J Nelson M Secreto C Warner SL Bearss DJ. Targeted Axl Inhibition Primes Chronic Lymphocytic Leukemia B Cells to Apoptosis and Shows Synergistic/Additive Effects in Combination With BTK Inhibitors. Clin Cancer Res (2015) 21(9):2115–26. doi: 10.1158/1078-0432.CCR-14-1892 PMC4479154 25673699

[74] Zhao GJ Zheng JY Bian JL Chen LW Dong N Yu Y. Growth Arrest-Specific 6 Enhances the Suppressive Function of CD4(+)CD25(+) Regulatory T Cells Mainly Through Axl Receptor. Mediators Inflamm (2017) 2017:13. doi: 10.1155/2017/6848430 PMC5320320 28270700

[75] Lodoen MB Lanier LL. Natural Killer Cells as an Initial Defense Against Pathogens. Curr Opin Immunol (2006) 18:391–8. doi: 10.1016/j.coi.2006.05.002 PMC7127478 16765573

[76] Galy A Travis M Cen D Chen B. Human T, B, Natural Killer, and Dendritic Cells Arise From a Common Bone Marrow Rogenitor Cell Subset. Immunity (1995) 3(4):459–73. doi: 10.1016/1074-7613(95)90175-2 7584137

[77] Di Santo JP. Natural Killer Cell Developmental Pathways: A Question of Balance. Annu Rev Immunol (2006) 24:257–86. doi: 10.1146/annurev.immunol.24.021605.090700 16551250

[78] Sceneay J Chow MT Chen A Halse HM Wong CS Andrews DM. Primary Tumor Hypoxia Recruits CD11b+/Ly6Cmed/Ly6G+ Immune Suppressor Cells and Compromises NK Cell Cytotoxicity in the Premetastatic Niche. Cancer Res (2012) 72:3906–11. doi: 10.1158/0008-5472.CAN-11-3873 22751463

[79] Caraux A Lu Q Fernandez N Riou S Di Santo JP Raulet DH. Natural Killer Cell Differentiation Driven by Tyro3 Receptor Tyrosine Kinases. Nat Immunol (2006) 7:747–54. doi: 10.1038/ni1353 16751775

[80] Smyth MJ Cretney E Kelly JM Westwood JA Street SEA Yagita H. Activation of NK Cell Cytotoxicity. Mol Immunol (2005) 42:501–10. doi: 10.1016/j.molimm.2004.07.034 15607806

[81] Screpanti V Wallin RP Grandien A Ljunggren HG. Impact of FASL-Induced Apoptosis in the Elimination of Tumor Cells by NK Cells. Mol Immunol (2005) 42:495–9. doi: 10.1016/j.molimm.2004.07.033 15607805

[82] Screpanti V Wallin RP Ljunggren HG Grandien A. A Central Role for Death Receptor Mediated Apoptosis in the Rejection of Tumors by NK Cells. J Immunol (2001) 167:2068–73. doi: 10.4049/jimmunol.167.4.2068 11489989

[83] Lieberman J. The ABCs of Granule-Mediated Cytotoxicity: New Weapons in the Arsenal. Nat Rev Immunol (2003) 3:361–70. doi: 10.1038/nri1083 12766758

[84] Trapani JA Smyth MJ. Functional Significance of the Perforin/Granzyme Cell Death Pathway. Nat Rev Immunol (2002) 2:735–47. doi: 10.1038/nri911 12360212

[85] Behrens EM Gadue PG Gong S-Y Garrett S Stein PL Cohen PL. The Mer Receptor Tyrosine Kinase: Expression and Function Suggest a Role in Innate Immunity. Eur J Immunol (2003) 33:2160–7. doi: 10.1002/eji.200324076 12884290

[86] Park I-K Giovenzana C Hughes TL Yu J Trotta R Caligiuri MA. The Axl/Gas6 Pathway is Required for Optimal Cytokine Signaling During Human Natural Killer Cell Development. Blood J Am Soc Hematol (2009) 113(11):2470–7. doi: 10.1182/blood-2008-05-157073 PMC2656272 18840707

[87] Waldmann TA Tagaya Y. The Multifaceted Regulation of Interleukin-15 Expression and the Role of This Cytokine in NK Cell Differentiation and Host Response to Intracellular Pathogens. Annu Rev Immunol (1999) 17:19–49. doi: 10.1146/annurev.immunol.17.1.19 10358752

[88] Suzuki H Duncan GS Takimoto H Mak TW. Abnormal Development of Intestinal Intraepithelial Lymphocytes and Peripheral Natural Killer Cells in Mice Lacking the IL-2 Receptor β Chain. J Exp Med (1997) 185:499–505. doi: 10.1084/jem.185.3.499 9053450 PMC2196040

[89] Biron CA Byron KS Sullivan JL. Severe Herpesvirus Infections in an Adolescent Without Natural Killer Cells. N Engl J Med (1989) 320:1731–35. doi: 10.1056/NEJM198906293202605 2543925

[90] Hafizi S Dahlback B. Signalling and Functional Diversity Within the Axl Subfamily of Receptor Tyrosine Kinases. Cytokine Growth Factor Rev (2006) 17:295–304. doi: 10.1016/j.cytogfr.2006.04.004 16737840

[91] Budagian V Bulanova E Orinska Z Thon L Mamat U Bellosta P. A Promiscuous Liaison Between IL-15 Receptor and Axl Receptor Tyrosine Kinase in Cell Death Control. EMBO J (2005) 24:4260–70. doi: 10.1038/sj.emboj.7600874 PMC1356322 16308569

[92] McKenna HJ Stocking KL Miller RE Brasel K De Smedt T Maraskovsky E. Mice Lacking Flt3 Ligand Have Deficient Hematopoiesis Affecting Hematopoietic Progenitor Cells, Dendritic Cells, and Natural Killer Cells. Blood (2000) 95:3489–97. doi: 10.1182/blood.V95.11.3489 10828034

[93] Yu H Fehniger TA Fuchshuber P Thiel KS Vivier E Carson WE. Flt3 Ligand Promotes the Generation of a Distinct CD34(+) Human Natural Killer Cell Progenitor That Responds to Interleukin-15. Blood (1998) 92:3647–57. doi: 10.1182/blood.V92.10.3647 9808558

[94] Hannum C Culpepper J Campbell D McClanahan T Zurawski S Bazan JF. Ligand for FLT3/FLK2 Receptor Tyrosine Kinase Regulates Growth of Haematopoietic Stem Cells and Is Encoded by Variant RNAs. Nature (1994) 368:643–8. doi: 10.1038/368643a0 8145851

[95] Lyman SD Brasel K Rousseau AM Williams DE. The Flt3 Ligand: A Hematopoietic Stem Cell Factor Whose Activities Are Distinct From Steel Factor. Stem Cells (1994) 12(Suppl1):99–107. discussion 108-110. 7535149

[96] Park I-K Trotta R Yu J Caligiuri MA. Axl/Gas6 Pathway Participates in Human Natural Killer Cell Development by Positively Regulating FLT3 Activation. Eur J Immunol (2013) 43:10. doi: 10.1002/eji.201243116 23722894 PMC3829002

[97] Scutera S Fraone T Musso T Cappello P Rossi S Pierobon D. Survival and Migration of Human Dendritic Cells Are Regulated by an IFN-Alpha-Inducible Axl/Gas6 Pathway. J Immunol (2009) 183(5):3004–13. doi: 10.4049/jimmunol.0804384 19657094

[98] Bennett MR. Apoptosis of Vascular Smooth Muscle Cells in Vascular Remodelling and Atherosclerotic Plaque Rupture. Cardiovasc Res (1999) 41(2):361–8. doi: 10.1016/S0008-6363(98)00212-0 10341835

[99] Melaragno MG Cavet ME Yan C Tai L-K Jin Z-G Haendeler J. Gas6 Inhibits Apoptosis in Vascular Smooth Muscle: Role of Axl Kinase and Akt. J Mol Cell Cardiol (2004) 37(4):881–7. doi: 10.1016/j.yjmcc.2004.06.018 15380678

[100] Melaragno MG Wuthrich DA Poppa V Gill D Lindner V Berk BC. Increased Expression of Axl Tyrosine Kinase After Vascular Injury and Regulation by G Protein-Coupled Receptor Agonists in Rats. Circ Res (1998) 83:697–704. doi: 10.1161/01.RES.83.7.697 9758639

[101] Pollman MJ Hall JL Gibbons GH. Determinants of Vascular Smooth Muscle Cell Apoptosis After Balloon Angioplasty Injury. Influence of Redox State and Cell Phenotype. Circ Res (1999) 84:113–21. doi: 10.1161/01.RES.84.1.113 9915780

[102] Yamada T Akishita M Pollman MJ Gibbons GH Dzau VJ Horiuchi M. Angiotensin II Type 2 Receptor Mediates Vascular Smooth Muscle Cell Apoptosis and Antagonizes Angiotensin II Type 1 Receptor Action: An *In Vitro* Gene Transfer Study. Life Sci (1998) 63:L289–95. doi: 10.1016/S0024-3205(98)00448-2 9806232

[103] Bai H Pollman MJ Inishi Y Gibbons GH. Regulation of Vascular Smooth Muscle Cell Apoptosis. Modulation of Bad by a Phosphatidylinositol 3-Kinase-Dependent Pathway. Circ Res (1999) 85:229–37. doi: 10.1161/01.RES.85.3.229 10436165

[104] Linger RMA Cohen RA Cummings CT Sather S Migdall-Wilson J Middleton DHG. Mer or Axl Receptor Tyrosine Kinase Inhibition Promotes Apoptosis, Blocks Growth and Enhances Chemosensitivity of Human non-Small Cell Lung Cancer. Oncogene (2013) 32:3420–31. doi: 10.1038/onc.2012.355 PMC3502700 22890323

[105] Scott RS McMahon EJ Pop SM Reap EA Caricchio R Cohen PL. Phagocytosis and Clearance of Apoptotic Cells Is Mediated by MER. Nature (2001) 411:207–11. doi: 10.1038/35075603 11346799

[106] Cohen PL Caricchio R Abraham V Camenisch TD Jennette J Roubey RAS. Delayed Apoptotic Cell Clearance and Lupus-Like Autoimmunity in Mice Lacking the C-Mer Membrane Tyrosine Kinase. J Exp Med (2002) 196:135–40. doi: 10.1084/jem.20012094 PMC2194017 12093878

[107] Rachel MA Linger AK Keating H Shelton E. Graham DK. TAM Receptor Tyrosine Kinases: Biologic Functions, Signaling, and Potential Therapeutic Targeting in Human Cancer. Adv Cancer Res (2008) 100:35–83. doi: 10.1016/S0065-230X(08)00002-X 18620092 PMC3133732

[108] Bemcentinib (BGB324) in Combination With Pembrolizumab in Patients With Advanced NSCLC. NIH. Available at: https://clinicaltrials.gov/ct2/show/NCT03184571 (Accessed Oct 04, 2021).

[109] Horn LA Fousek K Palena C. Tumor Plasticity and Resistance to Immunotherapy. Trends Cancer (2020) 6(5):432–41. doi: 10.1016/j.trecan.2020.02.001 PMC7192950 32348738

[110] Ye X Li Y Stawicki S Couto S Eastham-Anderson J Kallop D. An Anti-Axl Monoclonal Antibody Attenuates Xenograft Tumor Growth and Enhances the Effect of Multiple Anticancer Therapies. Oncogene (2010) 29:5254–64. doi: 10.1038/onc.2010.268 20603615

[111] Cerchia L Esposito CL Camorani S Rienzo A Stasio L Insabato L. Targeting Axl With an High-Affinity Inhibitory Aptamer. Mol Ther (2012) 20(12):2291–303. doi: 10.1038/mt.2012.163 PMC3519989 22910292

[112] Esposito C Catuogno S Franciscis V Cerchia L. New Insight Into Clinical Development of Nucleic Acid Aptamers. Discov Med (2011) 11(61):487–96. 21712014

[113] ClinicalTrials.gov. (Accessed Oct 04, 2021).

[114] Leconet W Larbouret C Chardès T Thomas G Neiveyans M Busson M. Preclinical Validation of AXL Receptor as a Target for Antibody-Based Pancreatic Cancer Immunotherapy. Oncogene (2014) 33:5405–14. doi: 10.1038/onc.2013.487 PMC5055582 24240689

